# Case report: A novel c.1842_1845dup mutation of *ETFDH* in two Chinese siblings with multiple acyl-CoA dehydrogenase deficiency

**DOI:** 10.3389/fped.2022.1038440

**Published:** 2023-01-04

**Authors:** Gaopin Yuan, Xiaohong Zhang, Tingli Chen, Jiansheng Lin

**Affiliations:** ^1^ Department of Endocrinology, Quanzhou Women's and Children's Hospital, Quanzhou, China; ^2^ Department of Laboratory Medicine, Quanzhou Women's and Children's Hospital, Quanzhou, China

**Keywords:** multiple acyl-CoA dehydrogenase deficiency, ETFDH, c.1842_1845dup, novel, mutation

## Abstract

This article reports the characterization of two siblings diagnosed with late-onset multiple Acyl-CoA dehydrogenase deficiency (MADD) caused by mutations in electron transfer flavoprotein(ETF)-ubiquinone oxidoreductase (ETF-QO) (*ETFDH*) gene. Whole exome sequencing (WES) was performed in the proband's pedigree. Clinical phenotypes of Proband 1 (acidosis, hypoglycemia, hypotonia, muscle weakness, vomiting, hypoglycemia, hepatomegaly, glutaric acidemia, and glutaric aciduria) were consistent with symptoms of MADD caused by the *ETFDH* mutation. However, Proband 2 presented with only a short stature. The patients (exhibiting Probands 1 and 2) showed identical elevations of C6, C8, C10, C12, and C14:1. c.1842_1845 (exon13)dup, and c.250 (exon3) G > A of the *ETFDH* gene were compound heterozygous variants in both patients. The novel variant c.1842_1845dup was rated as likely pathogenic according to the American College of Medical Genetics and Genomics guidelines (ACMG). This is the first report on the c.1842_1845dup mutation of the *ETFDH* gene in patients with late-onset MADD, and the data described herein may help expand the mutation spectrum of *ETFDH*.

## Introduction

Multiple Acyl-CoA dehydrogenation deficiency (MADD) is a rare, chromosomal, recessive, inherited metabolic deficiency that affects the metabolism of fatty acids, amino acids, and cholines (1). The incidence of neonatal MADD varies significantly by region and is approximately 1/299753 in China (2). MADD, also known as glutaric acidemia II or glutaric aciduria II (GAII), can be caused by mutations in any of alpha subunit ETF (*ETFA*) gene, beta subunit ETF (*ETFB*) gene and *ETFDH* gene. According to *ETFA*, *ETFB*, and *ETFDH* genes deficiencies, MADD can be subclassified as GAIIA, GAIIB, and GAIIC, respectively. The clinical presentation of MADD is highly heterogeneous, lacks specific symptoms and signs, and can develop at any time from the neonatal stage to adulthood (2). According to the age of onset, MADD can be divided into three types: Types I, II, and III. Types I and II have onset during the neonatal period, while Type III is typically associated with congenital malformations, such as polycystic kidney disease and midface hypoplasia. Type III, also known as late-onset MADD, generally occurs in infants to adults (2). Types I and II are more severe, often fatal, and characterized by nonketotic hypoglycemia, metabolic acidosis, and the accumulation and excretion of metabolites. In contrast, Type III is milder, with predominant involvement in skeletal muscle (3, 4); its symptoms are more variable and generally characterized by muscle involvement (myalgia and weakness), recurrent hypoglycemia, metabolic acidosis, and vomiting (5). Furthermore, some cases in China have been reported with an unusual neuropathy unresponsive to riboflavin (6). Late-onset MADD is diagnosed by a characteristic pattern of urine organic acids, demonstrating elevations of various combinations of aliphatic mono- and dicarboxylic acids, including 2-hydroxyglutaric, ethylmalonic, hippuric, adipic, and sebacic acids (7). In addition, a characteristic plasma acylcarnitine profile would show an increase in short-, medium-, and long-chain acylcarnitines (C4–C18:1) in neonatal screening using tandem mass spectrometry (2, 8). Here, we report a case of two siblings with late-onset MADD carrying a novel compound heterozygous mutation in *ETFDH*.

## Case description

### Proband 1

A nine-month-old boy chiefly complained of vomiting and mental fatigue; these symptoms were reported to have lasted for half a day by the parents. The patient presented the following recent illness history: the day before admission, the child vomited stomach contents twice without obvious triggers and subsequently experienced mental fatigue and hypoglycemia. After half a day of outpatient treatment, the child remained mentally fatigued, with a poor response and reduced appetite. His parents denied any dietary complications. The patient was transferred to our hospital for further diagnosis and treatment. Since disease onset, the patient had experienced mental fatigue, decreased appetite, and poor sleep, although no significant change was observed in body weight. The patient's health and personal history were as follows (1): Birth history: G2P2, full-term vaginal delivery; birth weight 3.85 kg. (2) Newborn disease screening history: An newborn screening sample obtained on the third day after birth was reported to be elevations of C0, C6, C8, C10, C12, C14:1, and C14:2. (3) Feeding history: Breastfed for four months and post-formula fed until admission, with the gradual addition of complementary food from six months of age with no adverse reactions. (4) Growth and developmental history: Weight and height gradually increased, with laughing at two months, stable neck erection at three months, and turning over at five months; however, unstable neck erection was observed after admission at nine months. (5) Family history: Healthy parents and non-consanguineous marriages, although the patient's sibling exhibited abnormal screening results, poor overall health, and a low height and weight and was prone to colds. Physical examination showed that the body weight was 9.0 kg; height was 72 cm; the limbs were hypotonia; the patient's neck erection was unstable. He was unable to sit alone because the lower limbs could not support his body weight in the standing position. However, bilateral knee tendon and bilateral Achilles tendon reflexes were elicited symmetrically. No abnormal touch was found, physiological reflexes were present, and Bruchner's, Klinefelter's, and double Barthel's signs were all negative. The preliminary diagnosis was a suspected genetic metabolic disease.

Auxiliary examination revealed the following: (1) Color Doppler ultrasound: Hepatomegaly, 5.0 cm below the right rib, liver parenchyma echo enhancement; (2) blood routine test: WBC, 27.1 × 10^*9^/l; (3) biochemical examination: HCO3^−^, 14.2 mmol/L; TBA, 51.6 µmol/L; ALT, 110 U/L; AST, 250 U/L; GLU, 2.90 mmol/L; UA, 550 µmol/L; LDH, 429 U/L; and CK, 222 U/L; (4) urine organic acids: Glutaric acid, 2-hydroxyglutaric acid, 3-hydroxyglutaric acid, and other glutaric acid increased; (5) blood mass spectrometry screening: C0, C6, C8, C10, C12, C14:1, and C14:2 increased; (6) the remainder of the examinations, such as brain MRI and ECG, were all normal, as shown in Table 1. To confirm the diagnosis, peripheral blood samples were collected from the proband for WES.

**Table 1:** Results of laboratory and imaging examination of proband 1 in first hospitalization and proband 2 in first visit.

Proband (no)	WBC (10^*9^/L)	FG (g/L)	HCO3-(mmol/L)	GLU (mmol/L)	ALT (U/L)	AST (U/L)	UA (µmol/L)	LDH (U/L)	CK (U/L)	Elevated Urine organic acids	Elevated blood acylcarnitine	Color Doppler Ultrasound	Brain MRI
1	27.1	0.6	17.2	2.9	110	250	550	429	222	Glutaric acid, 2-hydroxyglutaric acid, 3-hydroxyglutaric acid, etc.	C0, C6, C8, C10,C12,C14:1,C14:2	Hepatomegaly	Normal
2	ND	Normal	Normal	Normal	Normal	Normal	Normal	Normal	Normal	3–3-hydroxyphenyl-3-hydroxypropionic acid	C6,C8, C10, C10:1, C12, C14:1,C14:2	Normal	ND

Ref. Range: WBC, 5-1,210^*9^/L; FG, 2-4 g/L; HCO3-, 21-25 mmol/L; GLU, 3.9-6.1 mmol/L; ALT, 0-45 U/L; AST, 0-45 U/L; UA, 0–420 µmol/L; LDH, 109-245 U/L; CK, 18-198 U/L; ND, not detected.

After hospitalization, ceftazidime anti-infection, glutathione liver protection, and other treatments were all administered. According to the children's blood tandem mass spectrometry and urine organic acid results, late-onset MADD was considered a possibility, and in response, the patient was given large doses of vitamin B2 (50 mg, tid), levocarnitine (10 ml, oral tid), coenzyme Q10 (50 mg, tid). After 1 week, WES results confirmed our diagnosis. At the time of discharge, the patient appeared responsive and exhibited normative behavior for his age. However, the liver reached 3 cm below the ribs, and low muscle tone was observed in the limbs. Furthermore, unstable neck erection continued; the patient could not raise his head in the prone position, support the elbow, sit unsupported, and support the weight of the lower limbs in the standing position. Prescription of the doctor during discharge: Levocarnitine 3.33 ml, oral tid; vitamin B2 50 mg, tid; coenzyme Q10 50 mg, tid; compound glycyrrhizin half tablet tid; low-fat, low-protein, high-carbohydrate diet. Blood mass spectrometry and urinary organic acids were rechecked one week after hospitalization: C6, C8, C10, C10:1, C12, C14:1, and C14:2 all increased. Meanwhile, urinary organic acid results were normal.

After follow-up at six months, physical examination showed that body weight was 10.4 kg and height is 79 cm. He can sit alone, hold his head up, turn over, crawl, pronounce babamama, and walk a few steps alone. Muscle strength and muscle tone of the limbs were normal, physiological reflexes existed, and pathological signs were all negative. Auxiliary examination revealed C6, C8, C10, C10:1, C12, C14:1, and C14:2 all increased, however, urinary organic acids results remained normal.

After a subsequent follow-up at one year, physical examination showed that the weight was 11 kg, height was 88 cm, and he can walk alone and express simply. Blood tandem mass spectrometry showed that C6, C8, C10, C10:1, C12, C14:1, and C14:2 still increased.

### Proband 2

A 4-year and 1-month-old girl, the sister of Proband 1, first went to see a doctor because her younger brother was diagnosed with late-onset MADD, and she was usually in poor health, prone to colds, and showed low height and weight. Past history: Newborn screening results: Increased C6, C8, C10, C10:1, C12, C14, C14:1, and C14:2. Additionally, physical examination showed a weight of 13 kg (-SD: one standard deviation lower than average) and a height of 97.8 cm (-SD). However, bilateral knee tendon and bilateral Achilles tendon reflexes were elicited symmetrically, no abnormal touch was found, physiological reflexes were present, and Bruchner's, Klinefelter's, and double Barthel's signs were all negative. Auxiliary examination showed that C6, C8, C10, C10:1, C12, C14:1, and C14:2 increased, while urinary organic acid, 3–3-hydroxyphenyl-3-hydroxypropionic acid, also increased (Table 1). Proband 2 was initially diagnosed with MADD. To confirm this diagnosis, peripheral blood samples were drawn from the patient for WES. According to the WES test and other test results above, proband 2 was diagnosed with late-onset MADD. Treatment consisted of oral vitamin B2 50 mg tablets twice daily and oral coenzyme Q10 50 mg tablets twice daily.

A follow-up after six months revealed the following: (1) physical examination: Body weight was 14 kg (-SD), height was 100.2 cm (-SD); (2) auxiliary examination: Blood tandem mass spectrometry showed that C6, C8, C10, C10:1, C12, C14:1, and C14:2 increased, while urine organic acid analysis showed that 3–3-hydroxyphenyl-3-hydroxypropionic acid (23.44) increased.

An additional follow-up after one year revealed the following: (1) physical examination: Body weight was 14.8 kg (-SD), height was 105.1 cm (-SD); (2) auxiliary examination: Blood tandem mass spectrometry showed that C6, C8, C10, C10:1, C12, C14:1, and C14:2 increased, and urine organic acid analysis showed that 3–3-hydroxyphenyl-3-hydroxypropionic acid level was normal.

## Method

Trio WES was performed on four members of the family (both patients and their parents). Beijing Full Spectrum Medical Laboratory used chip capture high-throughput sequencing (IDT Company xGen® Exome Research Panel v2.0 capture probe) to detect the whole exon region of 20,000 genes in the human genome and then analyzed the diseases reported in OMIM, MedGen, and other databases based on the patient's clinical information to identify the genetic etiology. Whole family sequencing data quality control: target area coverage at least 99.60%, target area average depth >30× coverage at least 96.6%, target area data quality Q20 at least 0.95. The mutations were verified by Sanger sequencing.

## Results

Clinical phenotypes of Proband 1 (acidosis, hypoglycemia, hypotonia, muscle weakness, vomiting, hypoglycemia, hepatomegaly, glutaric acidemia, and glutaric aciduria) were consistent with symptoms of MADD caused by *ETFDH* mutation*.* A heterozygous missense mutation c.250 (exon3)G > A was found in *ETFDH* of the Proband 1, which resulted in *p*.A84T (*p*.Ala84Thr), as shown in Table 2. Supplementary Figure S1 shows that the mutation was validated by Sanger sequencing. Pedigree analysis revealed that the heterozygous mutation was inherited from the asymptomatic carrier mother. (Supplementary Figure S1). The minor genotype frequency of this mutation site is 0.0024 in 1,000 Genomes (China), which does not meet the PM2 standard of <0.0005. According to ACMG guidelines, the pathogenic evidence of this variant was “PM3_Very_Strong + PM1 + PP3″; and it was therefore rated as pathogenic. The other novel heterozygous frameshift mutation c.1842_1845 (exon13)dup was found in *ETFDH* of Proband 1, which resulted in *p*.G616fs*8 (*p*.Gly616fsTer8), as shown in Table 2. Supplementary Figure S2 shows the validation of this mutation using Sanger sequencing. The pathogenic evidence of this variant was “PVS1_Moderate + PM2 + PM3″; therefore, it was rated as likely pathogenic. PVS1_Moderate: Loss of function is a pathogenic mechanism that occurs at the 3′ end, affects no more than 10% of the total protein length, and is not expected to cause transcript degradation. PM2: This mutation is not included in any normal population databases. PM3: A pathogenic variant c.250 (exon3) G > A was detected in the trans position of Proband 1 in this study. Pedigree analysis revealed that the heterozygous mutation was inherited from the asymptomatic carrier father (Supplementary Figure S2). This compound heterozygous variant of *ETFDH* in Proband 1 fits the autosomal recessive inheritance pattern of MADD.

**Table 2:** Information on gene variant sites.

Gene	Chromosomal location	Rs number	Variant site	Types of mutation	Proband or	Father	Mother	Proband'sister	ACMG Pathogenicity rating	Related diseases (OMIM); inheritance pattern
*ETFDH*	chr4:15 9,603,421	Rs 200,309,328	NM_00445 3:c.250 (exo n3) G > A;*p*.A84T (*p*.Ala84Thr)	Missense	Heterozygous	Wild type	Heterozygous	Heterozygous	*P*	MADD (231,680); AR
	chr4:15 9,629,670–1,596,296 71	–	NM_00445 3:c.1842_1 845 (exon13) dup; *p*.G616fs*8 (*p*.Gly61 6fsTer8)	Frameshift	Heterozygous	Heterozygous	Wild type	Heterozygous	LP	

*P*, pathogenic; LP, likely pathogenic; AR, autosomal recessive.

This compound heterozygous variant (c.250G > A and c.1842_1845dup) in *ETFDH* was also found in Proband 2. The stature of Proband 2 was only somewhat lower than average.

## Discussion

MADD is a fatty acid oxidation disorder caused by electron transfer defects in the respiratory chain. Functional defects in either of ETF and ETF-QO result in electrons from the dehydrogenases of mitochondria beta-oxidation not being accepted, while C8–C14 levels are often elevated in children with late-onset MADD (2, 9). Furthermore, the majority of patients with MADD in Quanzhou showed elevations in C6, C8, C10, C12, C14, and C14:1 (10). Probands 1 and 2 showed identical elevations of C6, C8, C10, C12, and C14:1, and therefore, we believe that C6, C8, C10, C12, and C14:1 may be core markers of late-onset MADD. Intestinal clostridium could be involved in the pathogenesis of autism through 3–3-hydroxyphenyl-3-hydroxypropionic acid (11). There have not yet been any reports on the association between MADD and 3–3-hydroxyphenyl-3-hydroxypropionic acid. After the one-year follow-up, it was found that these elevations had finally returned to normal levels. However, this did not rule out the possibility that proband 2 had other nutritional and metabolic disorders.

Late-onset MADD may present within the first few months of life, later in childhood, or in adulthood, with hypoglycemia and acidosis or lipid storage myopathy and carnitine deficiency (12). This is consistent with the onset at nine months of age for Proband 1, who had clinical manifestations of hypoglycemia, acidotic myasthenia, and carnitine deficiency. In MADD, laboratory findings have an essential diagnostic role. Serum CK shows different levels of increase in patients, not correlated with the severity of muscle involvement or muscular pathology, and can be used as a biomarker of clinical progression. In cases where LDH is significantly higher than CK, clinicians can suspect MADD (13). We also found that in proband 1 serum CK was slightly higher, and serum LDH was much more higher than CK, which is consistent with its usefulness in the auxiliary diagnosis of MADD. We also found that the mild clinical presentation of Proband 2 with the same mutation was very different from that of Proband 1. A possible explanation for this could be that the phenotypic presentation of late-onset MADD is highly variable (14, 15), and the late-onset form usually has no obvious clinical symptoms (2). Mild short stature in Proband 2 may be a prodromal symptom of late-onset MADD.

Genetic testing is an important method for diagnosing MADD. Late-onset MADD cases are more common with *ETFDH* mutations, although few *ETFDH* mutations have been described in cases of neonatal-onset MADD (14, 16). c.250G > A in *ETFDH* is the most common mutation in late-onset MADD in China (17, 18). The carrier frequency of c.250G > A was estimated to be 1.35% in the normal population of southern China (17). The c.250G > A mutation can be used as a target for rapid screening of MADD in preimplantation genetic diagnosis and prenatal diagnosis. It is not surprising that we detected this mutation in this Chinese family. Additionally, the novel c.1842_1845dup frameshift mutation identified in this study enriches the pathogenic mutation spectrum of *ETFDH*.

Typically, patients with late-onset MADD can be treated using riboflavin and subsequently obtain good prognoses; hence, the late-onset form of MADD is known as riboflavin-responsive MADD (19). MADD patients are mainly treated with vitamin B2, L-carnitine, and coenzyme Q10 on a low-fat, high-carbohydrate, and moderate-protein diet. Most children with late-onset MADD exhibit obvious responses to therapeutic interventions, and early treatment can avoid, alleviate, or reverse clinical symptoms (20). Quality of life improved in Proband 1 after timely correction for metabolic dysregulation. Proband 2 was usually in poor health, prone to colds and showed a mild short stature, which may have been due to the overlooking of positive newborn screening results and lack of timely intervention. After treatment, the short stature of Proband 2 didn't significantly improved. Our findings emphasize the importance of early treatment in patients with late-onset MADD. There are some deficiencies in this study such as the lack of functional studies for mutations, and the possibility of a coexistent genetic/metabolic disease not identified by WES.

## Data Availability

The original contributions presented in the study are included in the article/**Supplementary Material**, further inquiries can be directed to the corresponding author/s.
