# Supplementary figures and images for: Case report: A novel c.1842_1845dup mutation of ETFDH in two Chinese siblings with multiple acyl-CoA dehydrogenase deficiency
Source: Front Pediatr. 2023 Jan 4;10:1038440. doi: 10.3389/fped.2022.1038440 (PMC9845722; doi:10.3389/fped.2022.1038440)

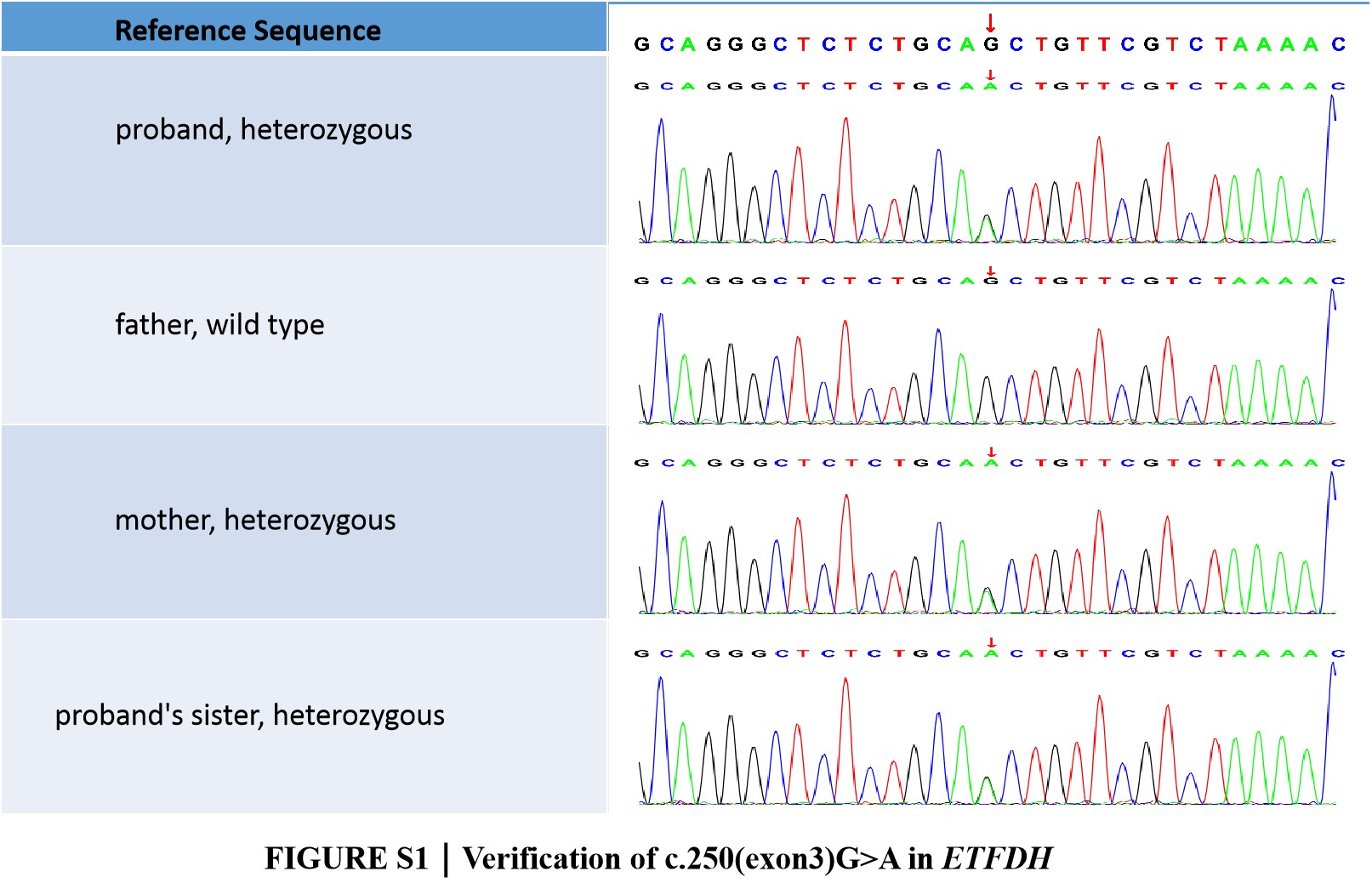

Supplement: Supplementary file 1 [file Image1.jpeg]

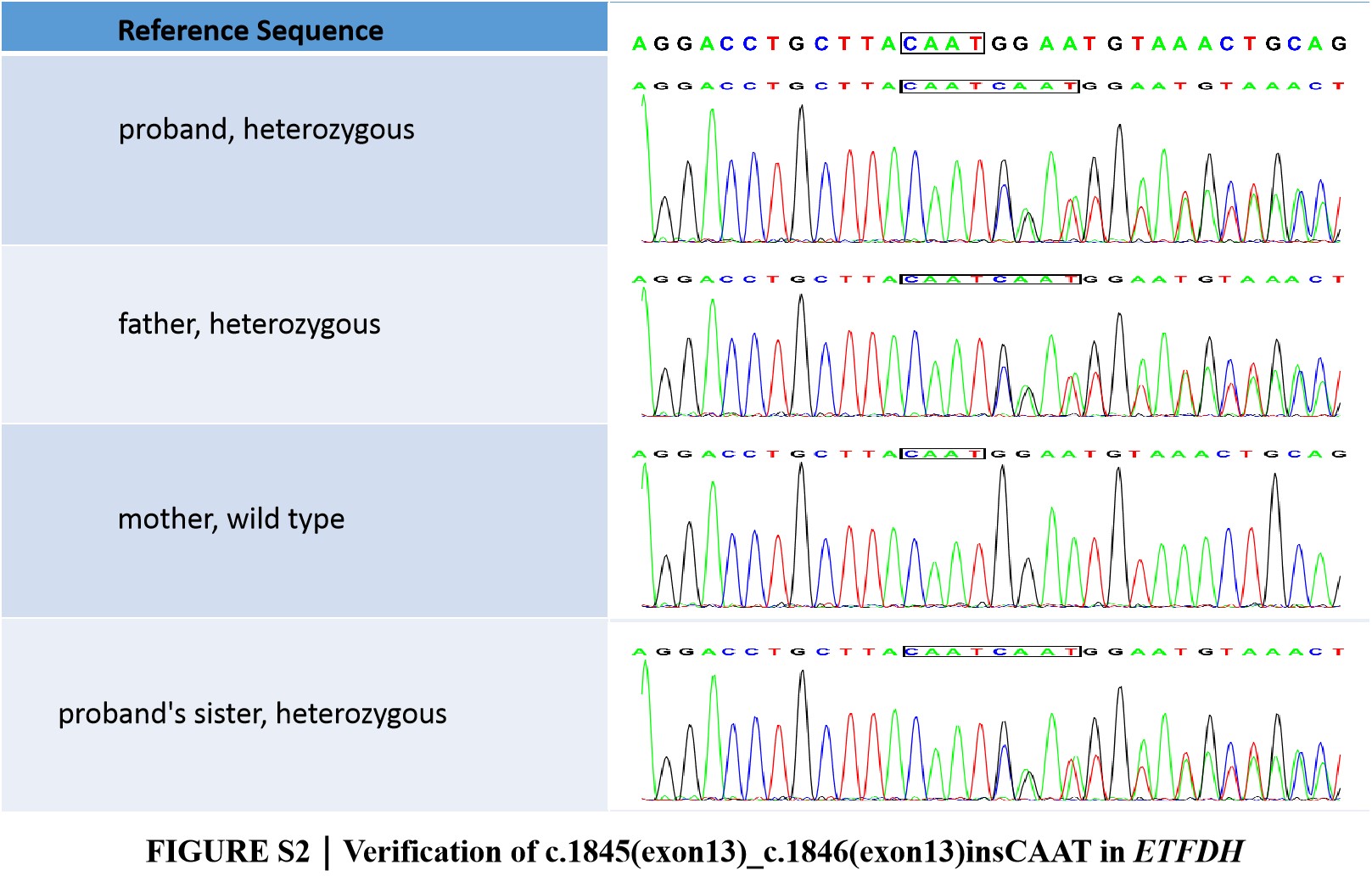

Supplement: Supplementary file 2 [file Image2.jpeg]
